# Case Report: Early onset pulmonary embolism following antipsychotic polypharmacy in an acute psychiatric setting

**DOI:** 10.3389/fpsyt.2026.1819958

**Published:** 2026-04-27

**Authors:** Stephan Fesenmeier, Martin Aigner, Dora Filipovits, Anna Höflich

**Affiliations:** Department of Psychiatry and Psychotherapeutic Medicine, University Hospital Tulln – NOE LGA, Karl Landsteiner University, Tulln, Austria

**Keywords:** adverse drug reaction, antipsychotic treatment, case report, drug safety, pulmonary embolism

## Abstract

Pulmonary embolism (PE) is a potentially life-threatening condition that may occur in patients receiving antipsychotic medication. Several studies have shown an increased risk of venous thromboembolism (VTE) associated with the use of antipsychotics, particularly during the first weeks or months of treatment. However, very early onset of VTE within hours of treatment initiation has been rarely reported. We present the case of a 69-year-old woman who developed acute pulmonary embolism within a few hours after initiation of psychopharmacological treatment for a psychotic episode. Due to acute agitation, the patient received olanzapine, quetiapine, levomepromazine, and lorazepam. The patient had no known predisposing risk factors for VTE. Shortly after begin of psychopharmacological treatment she presented with deterioration of her general condition, soporous, hypotensive, tachycardic and with reduced oxygen saturation. PE was suspected clinically and confirmed by computed tomography pulmonary angiography. After diagnosis, PE was successfully treated with anticoagulation. This case is notable for the unusually rapid development of VTE, occurring within hours rather than the typically reported days to weeks. It underscores the need for heightened clinical vigilance even in the very early phase of antipsychotic therapy and in patients without risk factors for VTE.

## Introduction

Acute pulmonary embolism (PE) is a common and potentially life-threatening cardiovascular emergency. The annual incidence of venous thromboembolism (VTE), including deep vein thrombosis and PE, is estimated at 117 cases per 100,000 persons (1). A substantial amount of VTE events are unprovoked: about one third to one half occur without any obvious underlying cause (2). Therefore, PE should be considered even when few risk factors are present.

Classic risk factors for VTE include increasing age, obesity, immobility (like surgery, trauma, hospitalization, long travel), malignancy, inherited thrombophilia, prior VTE (3) and pregnancy or oral contraceptive use and hormone therapy (4). In psychiatric patients additional risk factors like physical inactivity, obesity/metabolic syndrome, smoking, and comorbid medical conditions are more prevalent (5, 6). Catatonia, prolonged immobility and physical restraint in psychotic or depressive states are also thought to increase VTE risk. A chart review reported an incidence rate for deep vein thrombosis (DVT) of approximately 25% in catatonic inpatients (7) and another study detected DVT in 11.6% of restrained patients (8). These factors highlight the importance of risk assessment in clinical practice. The NICE guideline NG89 recommends assessment of venous thromboembolism and bleeding risk in all acute psychiatric inpatients (9), whereas a recent clinical practice guideline advises against routine pharmacological prophylaxis in low-risk scenarios, and recommends considering anticoagulant prophylaxis in high-risk patients when no contraindications are present (10).

Antipsychotic medication itself has repeatedly been associated with an increased risk of VTE. Several epidemiological studies and meta-analyses have shown a higher VTE risk in patients receiving antipsychotics compared with non-users (11–13). For example, in a large meta-analysis across 28 observational studies antipsychotic use was associated with higher odds of venous thromboembolism (OR 1.55) and pulmonary embolism (OR 3.68) relative to non-exposed individuals. Newly initiated users showed to be at higher risk (OR 2.06) (12). Another meta-analysis reported that low-potency antipsychotics had the highest VTE odds (OR. 2.91), with lower odds for second-generation (OR. 2.20) and high-potency neuroleptics (OR. 1.58) (13). First-generation agents like haloperidol and chlorpromazine and second-generation agents such as quetiapine have all been associated with VTE (14, 15). However, interpretation remains limited by scarce data and inconsistent findings across studies.

The biological mechanisms underlying the increased risk of venous thromboembolism in patients treated with antipsychotics are still unclear, and it is unlikely that a single mechanism explains this association. Several hypotheses have been proposed, including drug-related factors and illness-related factors (16). Antipsychotics may induce transient hyperprolactinemia, and prolactin is known to be a platelet co-activator, further contributing to coagulation (17).

Increased antiphospholipid antibodies (APLs) have been observed during antipsychotic therapy with Clozapine and first generation antipsychotics (18–20). The presence of elevated APLs increases VTE risk but similar elevations have also been found in psychotic patients without antipsychotic drug exposure (18).

Acute psychosis is also associated with elevated thrombogenesis markers, including platelet activation (P-selectin) and coagulation parameters (D-dimer, factor VIII), indicating that the disorder independently elevates VTE risk (21).

Overall, antipsychotics may increase the tendency for blood clot formation through several pathways. Furthermore, reduced mobility due to sedation plays an important role, as antipsychotics and other sedatives such as benzodiazepines can lead to immobility and decreased venous blood flow. In addition, weight gain and lipid changes related to atypical antipsychotics may increase procoagulant activity via inflammation pathways (22).

Most studies show that the risk of VTE is the highest within the first few months after initiation of antipsychotic treatment (15, 16, 23). However, despite this known association, there are only a few reports of PE occurring rapidly in an acute setting within hours. For example, in once case a patient developed PE 24 hours after hospitalization and initiation of Clozapine 25 mg. Notably, this patient had previously tolerated substantially higher clozapine doses without thromboembolic complications (24).

Our patient had no other predisposing conditions. We therefore present this case as an example of early-onset PE in an older patient receiving a combination of psychotropic medications due to a manic-psychotic clinical presentation, to raise awareness of this rare but potentially fatal complication.

## Case

A 69-year-old woman was acutely admitted to the psychiatric department of an university hospital because of threatening behavior, marked irritability, and an inability to care for herself. She had been previously hospitalized twice at the same department and was diagnosed with post-traumatic stress disorder and dissociative disorder. Apart from a history of strumectomy, she had no relevant somatic comorbidities. Her long-term outpatient medication consisted of sertraline 50 mg once daily, which, according to available information, had likely not been taken for several weeks prior to admission.

During initial psychiatric assessment, the patient presented herself with pressured speech, pronounced irritability and affectlability. Her thought process was disorganized and tangential, with prominent delusional content. Because of the severity of her symptoms and the associated risk of harming herself, involuntary hospitalization was initiated. Due to severe agitation and risk of harm, rapid tranquilization was required in accordance with clinical practice in acute psychiatric settings. Initial treatment with olanzapine 10 mg and lorazepam 2.5 mg was chosen to achieve both antipsychotic and anxiolytic/sedative effects. Because of insufficient response, additional medication was required and quetiapine 100 mg and levomepromazine 100 mg orally as well as lorazepam 2 mg intravenously were administered to enhance sedation and behavioral control.

Approximately two hours after medication administration, the patient’s general condition acutely deteriorated. She became soporous and was found to be severely hypotensive, with a blood pressure of 40/24 mmHg, increase of heart rate to 90/min, and a decrease of oxygen saturation to 90%. Emergency treatment with intravenous fluids and intravenous flumazenil was initiated, with insufficient clinical effect. Despite some hemodynamic stabilization, persistent hypoxemia was noted, with oxygen saturation remaining at approximately 85% without supplemental oxygen.

Repeated electrocardiographic examinations showed no evidence of sinus tachycardia, right heart strain, SIQIII pattern, or signs of ongoing myocardial ischemia. However, intermittent tachycardic episodes were observed on continuous monitoring. Given the high clinical suspicion of pulmonary embolism, computed tomography pulmonary angiography (CTPA) was performed. Imaging revealed multiple contrast filling defects in the peripheral pulmonary arteries of both lower lobes, as well as in the right apical and posterior upper lobes and the medial and lateral segments of the middle lobe. Additionally, broad-based consolidations in the posterior lower lobe were consistent with pulmonary infarction pneumonia.

Transthoracic echocardiography demonstrated no right ventricular dilatation, no signs of right ventricular pressure overload, and no evidence of pulmonary hypertension. Further diagnostic examination to identify potential underlying causes included abdominal ultrasound and chest radiography, both of which showed no pathological findings. Duplex ultrasonography of the lower extremities revealed no evidence of deep vein thrombosis. To exclude an underlying malignancy, upper and lower gastrointestinal endoscopy were performed. No suspicion of malignancy was identified. Small esophageal ulcerations were biopsied, and histological examination showed no malignant changes. Laboratory investigations at admission were largely unremarkable, except for a leukocytosis of 12 G/L and hypercholesterolemia. During the course of pulmonary infarction pneumonia, inflammatory markers increased, with a peak C-reactive protein (CRP) level of 4.9 mg/dL (normal range 0-0.5 mg/dL), which subsequently decreased to 1.6 mg/dL prior to discharge under antibiotic treatment with cefotaxime 2 grams twice daily.

Anticoagulation with low-molecular-weight heparin followed by dabigatran 150 mg twice daily was initiated in accordance with established treatment guidelines for acute pulmonary embolism.

From a psychiatric perspective, antipsychotic treatment with olanzapine was continued and titrated up to a total daily dose of 15 mg. Continuation of olanzapine was based on the patient’s favorable initial response and the need for effective control of acute psychotic symptoms. Under this regimen, the patient’s psychiatric symptoms improved rapidly.

By the time of discharge she appeared calm and cooperative, with coherent thought processes, no psychotic symptoms, and full insight into her illness. Consequently, involuntary hospitalization was changed to a voluntary status of admission. Due to daytime inactivity antipsychotic medication was later switched to aripiprazole, a less sedating antipsychotic, to improve functional recovery. At the time of discharge, the patient was still receiving olanzapine 7.5 mg daily, a further reduction during outpatient care was planned. The dose of aripiprazole was slowly titrated up to 15 mg daily during inpatient treatment. The patient was discharged in stable cardiopulmonary and psychiatric condition. The clinical course is summarized in **Table 1**.

**Table 1:** Timeline.

Time point	Clinical event	Medication/Intervention	Findings/Notes
Day 1 3:50 p.m.	Presentation with manic-psychotic symptoms		Acute psychiatric admission and examination
Day 1 4:00 p.m.		Acute medication, including olanzapine 10 mg and lorazepam 2.5 mg administered orally	
Day 1 7:03 p.m.	Persistent agitation and insufficient clinical response	Additional medication: quetiapine 100 mg and levomepromazine 100 mg administered orally and lorazepam 2 mg intravenously.	
Day 1 8:50 p.m.	Soporous; severe hypotension (40/24 mmHg), HR 90/min, SpO_2_ 90%	Intravenous fluids and intravenous flumazenil	Insufficient clinical effect
Day 1 9:10 p.m.	Clinical stabilization	Intravenous fluids	BP ~100/60 mmHg, HR ~80 bpm, SpO_2_ 100% on room air; arousable to pain
Day 1 9:22 p.m.	Electrocardiographic examination		No signs of sinus tachycardia, right heart strain or S1Q3T3 pattern
Day 2 -3	Patient presented as somnolent	Discontinuation of sedative medication.	
Day 3			Patient alert, responsive, oriented
Day 3	Continuous monitoring		Intermittent tachycardic episodes
Day 3	Computed tomography pulmonary angiography	Weight-adjusted low-molecular-weight heparin and antibiotic treatment with cefotaxime	Pulmonary embolism and infarction pneumonia
Day 4	Diagnostic workup: TTE, abdominal ultrasound, chest radiography, lower-limb duplex ultrasonography		No right ventricular dilatation or pulmonary hypertension on TTE, no deep vein thrombosis on duplex ultrasonography
Day 7 - 19		Reinitiation of Olanzapine; titrated to 15 mg/day.	Rapid symptom improvement
Day 17	Upper and lower GI endoscopy		No evidence of malignancy or other pathological findings on endoscopy
Day 20		LMWH transitioned to oral dabigatran 150 mg twice daily.	
Day 20	Sedation/daytime inactivity	Cross-tapering from olanzapine to aripiprazole	
Day 21	Hospital discharge		Calm and cooperative, coherent thought process, no psychotic symptoms, stable cardiopulmonary condition

## Discussion

This case is unusual due to the very rapid onset of PE within hours of starting therapy with a polypharmacy regimen of two atypical antipsychotics (olanzapine and quetiapine), one low-potency neuroleptic (levomepromazine), and a benzodiazepine (lorazepam). Previous studies and case reports have shown, that antipsychotic-associated PE typically developed within the first months of treatment (15, 25, 26). While individual agents like haloperidol, clozapine and quetiapine are associated with an increased VTE risk (14, 27) reports describing such an acute presentation within a day after treatment are rare. In prior studies there is a lack of detailed characterization of the temporal distribution within this early period. Specifically, current evidence does not specify how the risk for VTE evolves during the initial weeks and months of treatment, representing an important knowledge gap.

In our case we suspect a pharmacological effect of antipsychotic medication, reflected by the rapid clinical deterioration following drug administration. This case highlights important prescribing considerations in psychiatry, especially for older patients. Venous thromboembolism may develop shortly after the initiation of antipsychotic treatment rather than only after weeks or months, and this risk should be considered by clinicians. The patient also received lorazepam; however, any potential contribution to VTE would more plausibly be related to sedation-associated immobilization rather than a direct pharmacological effect. In this case, sedation was very brief and promptly reversed with flumazenil. A causal association between benzodiazepine exposure and VTE in this context therefore seems unlikely.

As age itself is a risk factor for VTE this 69 year old patient is at higher baseline risk. An assessment of VTE risk factors (history of thromboembolism, malignancy, obesity) should be a routine procedure before treating patients with such medications in less acute cases (9). Intermittent mobilization or prophylactic measures might be needed if older or medically ill patients require antipsychotic treatment. Furthermore, if hypotension or altered consciousness occurs in patients receiving psychotropic drugs, concern for acute medical complications such as pulmonary embolism should be raised after excluding unspecific effects caused by sedation. It is necessary that clinicians balance behavioral control against medical safety. This case urges extra caution when combining potent antipsychotic drugs in patients with increased VTE risk even during the first hours of treatment. Regular monitoring of vital signs including heart rate, respiratory rate, blood pressure, and oxygen saturation should be performed to support early detection of complications.

Diagnostically, this case shows us challenges on an acute psychiatric ward. The patient’s altered mental status and hypotension were immediately recognized as shock, but PE was initially not suspected. The absence of a calf swelling or DVT signs and no specific ECG presentation made diagnostic classification challenging. We did not obtain a D-dimer due to the emergency situation. In retrospect, an unclear sudden clinical deterioration, dyspnea, or chest pain in patients receiving multiple antipsychotic drugs should raise concern for pulmonary embolism. Therefore, close monitoring of respiratory rate, oxygen saturation and ECG is crucial.

It should also be noted that no thrombophilia testing was performed in this patient. Current evidence-based guidelines, including the 2023 recommendations of the American Society of Hematology, advise against routine thrombophilia testing in patients with unprovoked venous thromboembolism, as test results rarely influence acute management or the planned duration of anticoagulation (28).

In less acute cases, consultation with a clinical pharmacist may be beneficial for patients receiving antipsychotic polypharmacy during the admission and discharge processes to optimize prescribing practices (29). For example in a study from 2024, integrating a clinical pharmacist into ward rounds resolved 86.4% of manifest drug-related problems, achieved high recommendation acceptance (88.9% at discharge), and significantly improved guideline adherence (30). This is particularly relevant in elderly patients, who are frequently exposed to polypharmacy, a population that remains underrepresented in clinical trials despite its high prevalence in routine care (31).

## Conclusion

In conclusion, this case describes early pulmonary embolism following the initiation of combined olanzapine, quetiapine, levomepromazine and lorazepam treatment. It shows that vigilance for VTE is particularly important in acute psychiatric settings, which often lack routinely administered continuous monitoring. In patients treated with antipsychotics clinicians should inform high-risk patients about symptoms of VTE. Clinicians should be aware that venous thromboembolism can occur even shortly after the initiation of antipsychotic therapy. There should be a low threshold for diagnostic evaluation and anticoagulant treatment when thromboembolism is suspected, particularly in patients with predisposing factors. Further research is needed to better understand the prothrombotic effects of psychotropic medications and to develop strategies to reduce this risk and improve patient safety in mental health care. Furthermore, future guidelines should include recommendations regarding thrombosis prophylaxis in acutely treated psychiatric patients.

## Patient perspective

Initially, the patient described sedation with olanzapine and showed limited insight into the need for treatment. After cross-tapering to aripiprazole, she became more adherent and agreed to continue medication at discharge. She understood the diagnosis of pulmonary embolism and consented to anticoagulation. She described an increase of wellbeing at discharge.

## Data Availability

The datasets presented in this article are not readily available because due to patient privacy and regulation by the university clinic tulln, individual clinical data cannot be made publicly available. Requests to access the datasets should be directed to stephan.fesenmeier@tulln.lknoe.at.
